# Complete sputum smear monitoring among adults with pulmonary tuberculosis in central Uganda: evidence from a retrospective cohort study

**DOI:** 10.1186/s12879-022-07178-9

**Published:** 2022-02-25

**Authors:** Ronald Nsubuga, Norbert Adrawa, Stephen Okoboi, Alimah Komuhangi, Jonathan Izudi

**Affiliations:** 1 Institute of Public Health and Management, Clarke International University, Kampala, Uganda; 2 The AIDS Support Organization (TASO), Soroti Center of Clinical Excellence, Soroti, Uganda; 3 Infectious Diseases Institute (IDI), Makerere University College of Health Sciences, Kampala, Uganda; 4 Department of Community Health, Faculty of Medicine, Mbarara University of Science and Technology, Mbarara, Uganda

**Keywords:** Bacteriologically confirmed pulmonary tuberculosis, Smear-positive tuberculosis, Sputum smear monitoring, Uganda

## Abstract

**Background:**

People with bacteriologically confirmed pulmonary tuberculosis require sputum smear monitoring at 2, 5, and 6 months to establish treatment outcomes. However, there is limited information about sputum smear monitoring in Uganda, similar to other developing countries. We examined factors associated with complete sputum smear monitoring among persons with bacteriologically confirmed pulmonary TB aged ≥ 15 years in central Uganda.

**Methods:**

We retrospectively reviewed and abstracted data for persons with bacteriologically confirmed pulmonary TB initiated on treatment between January 2017 and December 2019 across 11 large TB units in Masaka district in central Uganda. Complete sputum smear monitoring was measured as the receipt of three sputum smear microscopy tests at 2, 5, and 6 months of TB treatment. The data were summarized descriptively and the differences in the outcome with independent variables were examined using tests of statistical significance, namely the Chi-square or Fisher’s exact test and the student’s t-test. The factors independently associated with the outcome were established using the modified Poisson regression analysis with robust standard errors, reported as adjusted risk ratio (aRR) along with the 95% confidence interval (CI).

**Results:**

A total of 416 participants were enrolled, with a mean age of 37.3 ± 12.9 years. Of the participants, 290 (69.7) were males, 269 (64.7) were rural residents, and 128 (30.8%) had complete sputum smear monitoring. Urban residence (aRR, 1.45; 95% CI 1.12–1.90) and treatment under the community-based directly observed therapy short-course strategy (DOTS) (aRR, 1.91; 95% CI 1.25–2.92) were associated with a higher likelihood of complete sputum smear monitoring while TB and human immunodeficiency virus (TB/HIV) comorbidity (aRR 0.45, 95% CI 0.30–0.68) was associated with a lower likelihood of complete sputum smear monitoring.

**Conclusions:**

We found a low magnitude of complete sputum smear monitoring among persons with bacteriologically confirmed pulmonary TB aged ≥ 15 years in central Uganda. Strategies to enhance the performance of sputum smear monitoring should target rural health facilities, strengthen TB/HIV collaboration and the implementation of community-based DOTS.

## Background

High morbidity and mortality posed by tuberculosis (TB) continue to be a global health problem despite the availability of effective treatment. The 2021 Global TB Report indicates that 9.9 million people fell ill with TB in 2020, with 1.3 million deaths among people who do not have human immunodeficiency virus (HIV) and an additional 214,000 deaths among people with HIV [1]. The African region still has the highest burden of TB [1]. In Uganda, approximately half of all persons diagnosed with pulmonary TB are bacteriologically confirmed [2], the most infectious form of TB disease. Persons with bacteriologically confirmed pulmonary TB pose an enormous risk of TB transmission at household and community levels especially when they sneeze, cough, laugh, spit, sing, talk, or breathe [3]. Therefore, proximity to these individuals puts one at an increased risk of TB infection, especially when they are not on treatment. The early diagnosis of pulmonary TB and initiation of treatment is advantageous in preventing onward transmission of TB. To establish a response to treatment, the Uganda National TB treatment guidelines recommend sputum smear monitoring at 2, 5, and 6 months [4], using sputum smear microscopy since GeneXpert and culture are expensive. GeneXpert is also unreliable because dead bacilli can still test positive even when response to treatment is good. At each of the visits, only one sputum specimen is required/collected. Based on sputum smear examination results, persons with a negative test continue with the first-line TB treatment while those with a positive test result receive GeneXpert to exclude rifampicin resistance. When an individual has a positive sputum smear monitoring test result but is rifampicin sensitive, treatment adherence counseling and support is provided while those found rifampicin resistant are started on second-line treatment for multi-drug resistant TB. Good response to treatment is reflected by sputum smear conversion, a change in sputum smear test result from positive to negative. Furthermore, sputum smear monitoring is useful in establishing treatment failure, the emergence of multi-drug resistance, and establishing cure [3].

Uganda has a suboptimal cure rate of 51% [5] and a treatment success rate of 78% according to the most recent national data [6]. These rates are far below the WHO desired targets of 85% cure rate and at least 90% treatment success rate [7]. One of the reasons for the suboptimal cure rate is a low level of complete sputum smear monitoring among persons with bacteriologically confirmed pulmonary TB [8], and incomplete sputum smear monitoring is equally a marker for suboptimal treatment success. However, information about complete sputum smear monitoring across most countries in sub-Saharan Africa is limited. Studies show that a substantial proportion of people with bacteriologically confirmed pulmonary TB do not complete sputum smear monitoring [9–12] despite its positive impact on treatment outcomes [8]. From literature, not being on directly observed therapy short-course strategy (DOTS) [11], being a male [10, 11], being unable to produce sputum, and experiencing longer waiting time at health facilities including inadequate patient health education on the importance, timing, and frequency of sputum smear monitoring [9, 11] are associated with incomplete sputum smear monitoring. We examined factors associated with complete sputum smear monitoring among people with bacteriologically confirmed pulmonary TB in Masaka district in central Uganda, a setting where program data show that the majority of persons with bacteriologically confirmed pulmonary TB do not complete sputum smear monitoring.

## Methods and materials

### Study design and setting

This retrospective cohort study involved the review of secondary data across 11 large government and private-not-for profit health facilities in Masaka district in central Uganda. In Uganda, the first level of healthcare is Health Center (HC) II at the parish level followed by HC III at the sub-county level, HC IV at the county level, district hospital at the district level, and then regional and national referral hospitals at the district and national levels, respectively.

Currently, there are five national referral hospitals, 14 regional referral hospitals, 169 general hospitals, 194 health center (HC) IVs, and the remaining are HC IIIs and HC. However, there are also five super-specialized hospitals in the country and two specialized institutes, the Uganda Heart Institute and the Uganda Cancer Institute [13].

Masaka district has 13 health facilities that provide TB diagnostic and treatment services (1 Regional Referral Hospital, 1 General Hospital, 2 HC IVs, and 7 HC IIIs) and 11 of these health facilities were included in the study because they have high patient load (100 TB patients diagnosed and treated per year, on average). The other two health facilities were not included but were used for pre-testing of the data abstraction tool because they have low patient load. Of the 11 study sites, the government health facilities included; Bukoto, Kamulegu, Bukeeri, Mpugwe, Buwunga, and Bukakata Health Center IIIs (HC IIIs), which are sub-county level health facilities. Also, Kyanamukaaka and Kiyumba HC IVs which are county-level health facilities, and Masaka Hospital, a regional level hospital were included. The private not-for-profit health facilities included Kitovu district hospital and Ssunga Health Centre III. Each of these study sites has a TB clinic that provides TB treatment and diagnostic services following the Uganda National TB and Leprosy Control Program (NTLP) Treatment guidelines. The diagnosis of drug-susceptible pulmonary TB using a GeneXpert occurs at HC IV and hospital levels of care, which are sites with a GeneXpert testing machine. At HC II level of care, only sputum smear microscopy is performed to diagnose TB. However, sputum smear samples are collected for examination at a GeneXpert site to exclude rifampicin drug resistance. People with bacteriologically confirmed pulmonary TB receive 6-months of anti-TB regimen in two phases. In the intensive phase, they receive 2 months of treatment that consist of Rifampicin (R), Isoniazid (H), Pyrazinamide (Z), and Ethambutol (E) administered as a fixed-dose combination of tablets. In the continuation phase, they receive RH for 4 months as a fixed-dose combination of tablets. Sputum smear monitoring is performed using microscopy at 2, 5, and 6 months of the treatment.

### Eligibility criteria

We included all persons aged ≥ 15 years diagnosed with bacteriologically confirmed pulmonary TB and initiated treatment between January 2017 and December 2019. We excluded the following categories of persons with TB: (1) persons whose response to TB treatment was not monitored using sputum smear microscopy, namely persons with clinically diagnosed and extrapulmonary TB; and, (2) persons whose sputum smear monitoring status could not be established, either because they were transferred to another health facility or were lost to follow-up.

### Study variables and measurements

Our outcome variable was complete sputum smear monitoring, measured on a binary scale. Persons who received all the three recommended sputum smear microscopy tests at 2, 5, and 6 months of TB treatment were regarded to have complete sputum smear monitoring and all the rest were regarded to have incomplete sputum smear monitoring. The independent variables included age measured in years and then categorized as 15–24, 25–34, 35–44, 45–54, and ≥ 55 years, sex (male versus female), TB/HIV comorbidity (no or yes), type of person with bacteriologically confirmed pulmonary TB namely new or retreatment case, availability of treatment supporter (no versus yes), place of residence (rural or urban), and type of DOTS (community-based versus health facility-based). The health facility attributes included the level of the health facility (health center versus hospital), type of health facility ownership (private-not-for profit versus government), and the location of the health facility, whether rural or urban.

### Data collection and processing

We abstracted data from TB unit registers using a standardized data abstraction tool between July and August 2020. Elsewhere [14], the dataset is deposited. Before data collection, the data abstraction tool was pre-tested outside the study area and the data collectors received training about the study purpose, data abstraction, and responsible conduct of research. All the filled data abstraction tools were reviewed for completeness before entry in Epi-Data [15]. To minimized data entry errors, quality control measures like skips, alerts, range, and legal values were employed in Epi-Data.

### Statistical data analysis

The analysis was done in Stata version 15 (Stata Corp., College Station, Texas, USA). Frequencies and percentages were computed for categorical data and means with standard deviations for numerical data at the univariate analysis. Complete sputum smear monitoring was computed as the proportion of participants with three sputum smear microscopy examinations scheduled at 2, 5, and 6 months of TB treatment.

In the bivariate analysis, the Chi-square test was employed to test differences in complete sputum smear monitoring with categorical data when all the cell counts were ≥ 5, otherwise, Fisher’s exact test was used. Mean differences in complete sputum smear monitoring with numerical data were compared with the Student’s t-test. The multivariate analysis included all variables with probability value (p-value) less than 5% at bivariate analysis and significant variables from the literature particularly residence. The outcome was frequent so the odds ratio would overestimate the degree of association [16]. Accordingly, we computed crude risk ratio (RR) and adjusted risk ratio (aRR) at multivariate analysis using the modified Poisson regression analysis with robust standard errors [17], and reported RR with the corresponding 95% confidence interval (CI).

We did not report probability values (p-values) since they are less informative [18]. CIs are sufficient for reporting the precision of the measure of effect and establishing statistical significance [18]. Additionally, CI is more informative than p-values [19] and is hence preferred in reporting the measure of effect [20–22].

### Ethical considerations

Our study was reviewed and approved by the Clarke International University Research Ethics Committee (reference number CLARKE-2020-31). Administrative approval was obtained from the Masaka District Health Office.

## Results

### Study profile

**Fig. 1:**
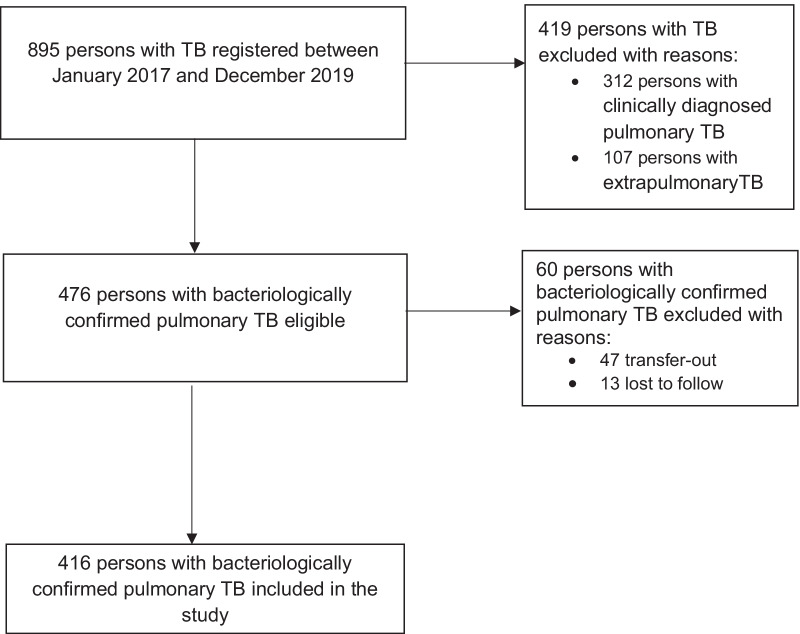
Study profile for complete sputum smear monitoring among persons with bacteriologically confirmed pulmonary TB in Masaka district in central Uganda

There were 895 persons with TB registered for treatment across the study sites between January 2017 and December 2019 (Fig. 1). Of this, a total of 479 persons were excluded with reasons: (1) 419 participants were ineligible because their response to treatment was monitored through clinical improvements but not sputum smear microscopy; and, (2) 60 participants were persons whose sputum smear status could not be established because they were either transferred to another health facility (n = 47) or were lost to follow-up (n = 13). Overall, we analyzed data for 416 participants.

### Distribution of participant characteristics

The mean age of the 416 participants was 37.3 ± 12.9 years, with a range of 15–82 years (Table 1). Most of the participants (36.1%) were in the 25–34 age category, males (69.7%), and persons with a new diagnosis of bacteriologically confirmed pulmonary TB (95.7%). Over half (65.6%) had received treatment under the community-based DOTS and 64.4% resided in a different sub-county to that of the health facility. Table 1 further shows that 64.7% of the participants were rural residents, less than half (41.6%) were persons with TB/HIV (41.6%), and the majority (81.0%) had a treatment supporter.

**Table 1:** Participant and health facility characteristics

Variable	Level	Overall (n = 416)
Health facility location	Rural	137 (32.9)
	Urban	279 (67.1)
Health facility level	Health center	137 (32.9)
	Hospital	279 (67.1)
Type of health facility ownership	Private-not-for profit	21 (5.1)
	Government	395 (95.0)
Age group (years)	15–24	52 (12.5)
	25–34	150 (36.1)
	35–44	104 (25.0)
	45–54	63 (15.1)
	≥ 55	47 (11.3)
	Mean	37.3 ± 12.9
Sex	Female	126 (30.3)
	Male	290 (69.7)
Type of person with BC-PTB	Retreatment	18 (4.3)
	New	398 (95.7)
Type of DOTS	Facility-based	143 (34.4)
	Community-based	273 (65.6)
Lives in the same sub-county as the health facility	No	268 (64.4)
	Yes	148 (35.4)
Residence	Rural	269 (64.7)
	Urban	147 (35.3)
TB/HIV	No	243 (58.4)
	Yes	173 (41.6)
Treatment supporter	No	79 (19.0)
	Yes	337 (81.0)

### Level of complete sputum smear monitoring and bivariate analysis of differences incomplete sputum smear monitoring with particiant and health facility factors

Of the 416 participants enrolled, 128 (30.8%) had complete sputum smear monitoring (Table 2). Participants with complete sputum smear monitoring were slightly older compared to those with incomplete sputum smear monitoring: 39.0 ± 14.2 versus 36.5 ± 12.2 (*p* = 0.067). The majority of participants with complete sputum smear monitoring were from urban health facilities, had received treatment at the hospital level of care, and a government health facility. Both females and males had a comparable proportion of complete sputum smear monitoring: 26.2% versus 32.8% respectively, *p* = 0.182. We found statistically significant differences in complete sputum smear monitoring concerning the type of health facility ownership (*p* = 0.006), type of DOTS (*p* < 0.001), TB/HIV co-infection (*p* < 0.001), and treatment supporter availability (*p* = 0.048).

**Table 2:** Bivariate analysis of differences in complete sputum smear monitoring with participant and health facility characteristics

Variable	Level	Overall (n = 416)	Complete sputum smear monitoring		P-value
			No (n = 288)	Yes (n = 128)	
Health facility location	Rural	137	101 (73.7)	36 (26.3)	0.164
	Urban	279	187 (67.0)	92 (33.0)	
Health facility level	Health center	137	101 (73.7)	36 (26.3)	0.164
	Hospital	279	187 (67.0)	92 (33.0)	
Type of health facility ownership	Private-not-for profit	21	20 (95.2)	1 (4.8)	0.006
	Government	395	268 (67.8)	127 (32.2)	
Age group (years)	15–24	52	38 (73.1)	14 (26.9)	0.368
	25–34	150	108 (72.0)	42 (28.0)	
	35–44	104	70 (67.3)	34 (32.7)	
	45–54	63	45 (71.4)	18 (28.6)	
	≥ 55	47	27 (57.4)	20 (42.6)	
	Mean	37.3 ± 12.9	36.5 ± 12.2	39.0 ± 14.2	0.067
Sex	Female	126	93 (73.8)	33 (26.2)	0.182
	Male	290	195 (67.2)	95 (32.8)	
Type of person with BC-PTB	Retreatment	18	9 (50.0)	9 (50.0)	0.071
	New	398	279 (70.1)	119 (29.9)	
Type of DOTS	Facility-based	143	120 (83.9)	23 (16.1)	< 0.001
	Community-based	273	168 (61.5)	105 (38.5)	
Lives in the same sub-county as the health facility	No	268	177 (66.0)	91 (34.0)	0.058
	Yes	148	111 (75.0)	37 (25.0)	
Residence	Rural	269	191 (71.0)	78 (29.0)	0.289
	Urban	147	97 (66.0)	50 (34.0)	
TB/HIV	No	243	141 (58.0)	102 (42.0)	< 0.001
	Yes	173	147 (85.0)	26 (15.0)	
Treatment supporter	No	79	62 (78.5)	17 (21.5)	0.048
	Yes	337	226 (67.1)	111 (32.9)	

### Factors associated with the complete sputum smear monitoring

Table 3 shows both the unadjusted and adjusted analyses findings. In the unadjusted analysis, community-based DOTS was associated with a higher likelihood of complete sputum smear monitoring (RR 2.39, 95% CI 1.60–3.58) while TB/HIV co-infection was associated with a lower likelihood of complete sputum smear monitoring (RR, 0.36; 95% CI 0.24–0.53). However, having a treatment supporter (RR, 1.53; 95% CI 0.98–2.40), living in the same sub-county as the health facility (RR, 0.74; 95% CI 0.53–1.02), and being an urban resident (RR, 1.17; 95% CI 0.87–1.57) were not associated with complete sputum smear monitoring. After adjusting for statistically significant and socially relevant variables namely residence and treatment supporter availability (Table 2), our data show that complete sputum smear monitoring was 45% more likely among urban residents compared to rural residents (aRR 1.45 95% CI 1.12–1.90) and when treatment had occurred under community-based DOTS compared to facility-based DOTS (aRR 1.91, 95% CI 1.25–2.92). Conversely, persons with TB/HIV were 55% less likely to complete sputum smear monitoring (aRR 0.45, 95% CI 0.30–0.68). However, having a treatment supporter was not associated with complete sputum smear monitoring (aRR 1.21, 95% CI 0.78–1.88).

**Table 3:** Factors associated with complete sputum smear monitoring at unadjusted and adjusted analysis

		Modified Poisson regression analysis			
Variable	Level	Unadjusted analysis		Adjusted analysis	
		RR	95% CI	aRR	95% CI
Lives in the same sub-county as the health facility	No	1			
	Yes	0.74	(0.53–1.02)		
Residence	Rural	1		1	
	Urban	1.17	(0.87–1.57)	1.45*	(1.12–1.90)
Type of DOTS	Facility-based	1			
	Community-based	2.39***	(1.60–3.58)	1.91**	(1.25–2.92)
Persons with TB/HIV	No	1		1	
	Yes	0.36***	(0.24–0.53)	0.45***	(0.30–0.68)
Health facility ownership	Private-not-for Profit	1			
	Government	6.75	(0.99–45.96)		
Treatment supporter	No				
	Yes	1.53	(0.98–2.40)	1.21	(0.78–1.88)

1) Statistical significance codes at 5%: *p < 0.05, **p < 0.01, ***p < 0.001; 2) DOTS: Directly observed therapy short course strategy; 3) RR: Unadjusted risk ratio; 4) aRR: Adjusted risk ratio

## Discussion

Our study focused on complete sputum smear monitoring among persons with bacteriologically confirmed pulmonary TB in central Uganda. We found that one-third of persons with bacteriologically confirmed pulmonary TB complete sputum smear monitoring, with completion being more likely among urban than rural residents and when treatment is under community-based DOTS compared to facility-based DOTS, but less likely among persons with TB/HIV comorbidity. The level of complete sputum smear monitoring in the present study is much lower than previously reported in Rwanda at 49.9% [12] and across two rural districts in Uganda at 45% [11] but comparable to 27.7% observed in a recent study in rural eastern Uganda [9].

In general, the magnitude of complete sputum smear monitoring remains distant from the Uganda Ministry of Health target of 100% (all persons with bacteriologically confirmed pulmonary TB receiving three sputum smear microscopy examinations) [4]. The lower magnitude of complete sputum smear monitoring undermines efforts to ascertain response to treatment, detect treatment failure, and drug resistance. This will exacerbate TB morbidity and mortality at both the household and community levels since persons with bacteriologically confirmed pulmonary TB infect on average 10–15 persons per year [23]. Given that TB control programs in sub-Saharan Africa have a suboptimal treatment success rate [24] and the positive impact of complete sputum smear monitoring on cure and treatment success rates [8], sputum smear monitoring should be a top priority for both the district and national TB control programs.

Our study shows that urban residents are more likely to complete sputum smear monitoring compared to rural residents. This could be due to easy access to health services in urban areas compared to rural areas. This finding is consistent with previous studies which report sputum smear monitoring is less likely among rural residents compared to urban residents [9, 25]. Accordingly, direct and indirect costs associated with access to TB care tend to negatively affect the continuity of care [26]. It is important to recognize that in Uganda, access to health facilities continue to be a challenge in rural areas despite the availability of the Uganda National Health Policy framework which aims to achieve universal access to basic health care package within a 5 km radius [27]. To date, the majority of the population lives ≥ 5 km from a health facility making access to healthcare difficult [28]. Accordingly, the Uganda government decentralized the delivery of health services by using Village Health Teams, the lowest level of the formal health system. However, uptake of TB services in rural areas is still challenging [16]. This is because, at the lowest level of healthcare, multiple operational challenges such as low motivation, inadequate funding, and a low level of community involvement are prevalent [14, 17]. Consequently, sputum smear monitoring appears not to be a priority. Another reason is inadequacies in staffing, supplies, reagents, and equipment to enable rural health facilities to perform follow-up sputum smear microscopy examinations [9].

Our finding of a higher likelihood of complete sputum smear monitoring under the community-based DOTS is not surprising. This is because DOTS enables supervised TB treatment and ensures compliance with treatment recommendations [29]. Data from systematic reviews and meta-analysis show that the community-based DOTS is associated with better treatment outcomes namely treatment success rate [30, 31], higher treatment completion rate, and lower mortality and transfer-out rates compared to the facility-based DOTS [31]. Our finding hence reinforces the need to strengthen the implementation of the community-based DOTS. Furthermore, the community-based DOTS allows the involvement of community support in TB treatment by establishing a community-based support system to enable treatment compliance and sputum smear monitoring [32]. Conversely, facility-based DOTS require individuals to visit the health facility on daily basis supervised treatment by a healthcare worker. This is logistically inefficient and ineffective given that persons with TB face physical and economic barriers [9].

Our data show that persons with TB/HIV are less likely to complete sputum smear monitoring. TB and HIV are overlapping epidemics hence healthcare providers must provide comprehensive information concerning the management of the comorbidity [18]. The present finding might be explained by an interplay of myriad factors like frequent TB/HIV clinic visits which are associated with both direct and indirect costs that compromise the continuity of care [33]. Also, persons with TB/HIV tend to have high levels of stigma which negatively impact compliance to treatment guidelines or schedules [34].

TB and HIV-related stigma combined with other factors like long travel distances equally influence compliance to treatment recommendations [34] and potentially results in missed appointments hence lost opportunity to monitor treatment response through sputum smear examinations. Our findings emphasize the need to strengthen TB/HIV collaborative activities to achieve better co-management and treatment outcomes.

## Study strengths and limitations

Our study has several strengths. First, few studies [9–12] have focused on complete sputum smear monitoring in sub-Saharan Africa. Therefore, our study presents additional information about complete sputum smear monitoring among persons with bacteriologically confirmed pulmonary TB in sub-Saharan Africa. We analyzed data from all the large TB units so the findings are likely generalizable to the setting and other similar settings in Uganda and elsewhere. However, there are limitations to consider. The analysis of secondary data is limited by the number of potential variables that influence complete sputum smear monitoring namely levels of education, counseling, income, and employment status among others. There is a possibility of missing or inaccurate data entries in the registers although attempts were made to verify sputum smear monitoring status using the TB laboratory register. The lack of qualitative data to explain the reasons for incomplete sputum smear monitoring from the perspectives of the participant, treatment supporter, and healthcare provider is another limitation to consider. Also, there is a possibility of selection bias by excluding lost to follow-up and transferred out participants especially if they have different characteristics from the included participants. However, it was logistically not possible to trace these participants and to establish their sputum smear monitoring status. We did not collect data on treatment outcomes so its relationship with complete sputum smear monitoring was not assessed.

## Conclusions and recommendations

Our study shows that among persons with bacteriologically confirmed pulmonary TB aged ≥ 15 years in central Uganda, the magnitude of complete sputum smear monitoring is low. Urban residence and treatment under community-based DOTs are associated with a higher likelihood of complete sputum smear monitoring while TB/HIV co-infection is associated with a lower likelihood of complete sputum smear monitoring. There is therefore a need to improve the performance of sputum smear monitoring in rural health facilities by addressing existing barriers and strengthening TB/HIV collaborative activities including the implementation of community-based DOTS.

## Data Availability

The datasets generated during and/or analysed during the current study are available in the Dryad repository, accessible at https://datadryad.org/stash/share/5uRuH_NhN6SsBQkDFCW5RlYoYP0TCFJACGzS8uCxPzo.

## Abbreviations

aRR: Adjusted risk ratio
DOTS: Directly Observed Therapy Short Course
HIV: Human Immunodeficiency Virus
RR: Unadjusted risk ratio
